# Ash leaf metabolomes reveal differences between trees tolerant and susceptible to ash dieback disease

**DOI:** 10.1038/sdata.2017.190

**Published:** 2017-12-19

**Authors:** Christine M. Sambles, Deborah L. Salmon, Hannah Florance, Thomas P. Howard, Nicholas Smirnoff, Lene R. Nielsen, Lea V. McKinney, Erik D. Kjær, Richard J. A. Buggs, David J. Studholme, Murray Grant

**Affiliations:** 1 Biosciences, Geoffrey Pope Building, University of Exeter, Stocker Road, Exeter EX4 4QD, UK; 2 School of Life Sciences, Gibbet Hill Campus, University of Warwick, Coventry CV4 7AL, UK; 3 SynthSys, Roger Land Building, Alexander Crum Brown Road, The King’s Buildings, Edinburgh EH9 3FF, UK; 4 School of Biology, Devonshire Building, Newcastle University, Newcastle upon, Tyne NE1 7RU, UK; 5 Department of Geosciences and Natural Resource Management, University of Copenhagen, Rolighedsvej 23, Frederiksberg C 1958, Denmark; 6 Royal Botanic Gardens Kew, Richmond, Surrey TW9 3AB, UK; 7 School of Biological and Chemical Sciences, Queen Mary University of London, Mile End Road, London E1 4NS, UK

**Keywords:** Metabolomics, Cheminformatics, Forestry, Natural variation in plants

## Abstract

European common ash, *Fraxinus excelsior*, is currently threatened by Ash dieback (ADB) caused by the fungus, *Hymenoscyphus fraxineus*. To detect and identify metabolites that may be products of pathways important in contributing to resistance against *H. fraxineus*, we performed untargeted metabolomic profiling on leaves from five high-susceptibility and five low-susceptibility *F. excelsior* individuals identified during Danish field trials. We describe in this study, two datasets. The first is untargeted LC-MS metabolomics raw data from ash leaves with high-susceptibility and low-susceptibility to ADB in positive and negative mode. These data allow the application of peak picking, alignment, gap-filling and retention-time correlation analyses to be performed in alternative ways. The second, a processed dataset containing abundances of aligned features across all samples enables further mining of the data. Here we illustrate the utility of this dataset which has previously been used to identify putative iridoid glycosides, well known anti-herbivory terpenoid derivatives, and show differential abundance in tolerant and susceptible ash samples.

## Background & Summary

The globalisation of trade and logistical biosecurity challenges at port of entry has led to an increasing number of alien species invading countries where they have often adapted to new environments and infected exotic flora. Furthermore, climate change is likely to modify plant pathogen profiles, further contributing to emerging pathogens^1^. Most visible and socially impactful are tree pathogens, which have the potential to dramatically modify the landscapes of countries or even continents. Recent examples include Dutch elm disease which began in the UK and chestnut blight in the USA^2^. Today, European common ash (*Fraxinus excelsior*) is currently threatened by Ash dieback (ADB) which was first reported in the early 1990’s in north-eastern Poland^3^ and from where it has rapidly spread across Europe^4–8^. ADB, caused by the fungus *Hymenoscyphus fraxineus*, is currently found in most European countries, and was confirmed in the UK and Ireland in 2012. In the UK alone, where in excess of 100 million trees are at threat, it is estimated that over 1,000 species rely on ash trees for all or part of their lifecycle, including wood mice, squirrels, bullfinches, wrens, bats and beetles. Forty five of these species are considered obligate^9^. The causal agent of ash dieback, *H. fraxineus* most probably originated from East Asia. Hosoya *et al.*^10^ reported the presence of the fungus^10,11^ in Japan on its native host Manchurian ash (*Fraxinus mandshurica*) where it exhibits a hemi-biotrophic lifestyle^12,13^. By contrast, on a range of exotic hosts, not just exclusively *Fraxinus* spp. but extending into other Oleaceae, *H. fraxineus* displays a necrotrophic lifestyle. Interestingly, emerald ash borer (*Agrilus planipennis*), a native Chinese buprestid beetle, has devastated tens of millions of *Fraxinus americana* (American ash) in the USA^14^. Yet, both *A. planipennis* and *H. fraxineus* appear to co-exist with Manchurian ash in their native habitats.

The recent discovery of a genetic basis to ADB susceptibility provides the opportunity for selective breeding approaches^15–18^ that will facilitate the identification and propagation of superior ash trees. Tree breeding programmes have been established to select tolerant/resistant germplasm. Nevertheless, such programmes take many years to establish, ideally require access to replicated field trials, and need large breeding population sizes to avoid loss of rare alleles and maximisation of long-term commercial traits and may not reveal the genetic background behind the resistance. An ideal solution would be to have robust markers to assist in selective breeding for ADB and recent progress on whole genome sequencing^19^ and association transcriptomics^20^ will facilitate identification of molecular markers for assisted ash breeding and help provide a molecular understanding of the tolerance identified by field testing. However, these studies are confounded by the high degree of genetic heterozygosity due to extensive outcrossing, making the identification of genetic markers challenging. Moreover, breeding value assessments of Danish tolerant ‘Tree 35’ concluded that resistance was quantitative and that there was no evidence to suggest that resistance to ADB operating in *F. excelsior* was a consequence of a single or a few resistance genes (qualitative resistance), thus race specificity is unlikely^21^.

Despite the extensive genetic heterogeneity in European *F. excelsior*, ADB tolerant trees are increasingly being reported across Europe, e.g., Havrdová *et al.*^22^ and Muñoz *et al.*^23^. Given (i) the evidence for quantitative resistance, (ii) that resistance mechanisms of deciduous trees are thought to include a combination of constitutive and induced chemical defences^21^ and (iii) as wind borne ascospores are present for a sustained period over the summer, the mechanism underpinning foliar infection tolerance to ADB is highly likely to have a major constitutive component. Based upon this knowledge we undertook an unbiased global metabolic profiling of ash. We used material from the highly advanced Danish study that identified ‘Tree 35’^21^. We first tested whether the methodological approach could discriminate tolerant and susceptible ash. Then, using a second independent set of individuals, we undertook a detailed metabolomics profiling of these Danish ash. Here we provide our untargeted LC-MS metabolomics raw data from ash trees with high-susceptibility and low-susceptibility to ADB, a processed dataset containing abundances of aligned features across all samples, and use this dataset to highlight a family of small molecules from the iridoid glycoside class, that discriminate tolerant and susceptible ash. We demonstrate that iridoid glycosides, well known antifeedant molecules, are markedly reduced in tolerant ash leaves. The ecological and breeding implications of this is intriguing, as it implies breeding for ADB tolerance may unintentionally confer enhanced susceptibility to emerald ash borer. Given its presence in Russia, and hence representing an emergent threat to Europe, these findings certainly warrant further investigation.

Our study provides a wealth of potential information for future investigation and are the only set of replicated, unbiased LC-MS metabolite data from verified tolerant and susceptible European ash and these are available with associated metadata in Metabolights^24,25^ [MTBLS372]. Thus different data processing algorithms can be applied to reuse these data. As a striking 25% of the ash genome encoded unique (orphan) genes^19^, we see genuine utility in using this dataset to facilitate metabolite predictions to support studies aimed at identifying gene function. While we have focussed on the iridoid glycosides, the processed dataset contains features that discriminate tolerant trees and may be developed into rapid screens for ADB tolerant ash, e.g., in breeding trials.

## Methods

**Figure 1:**
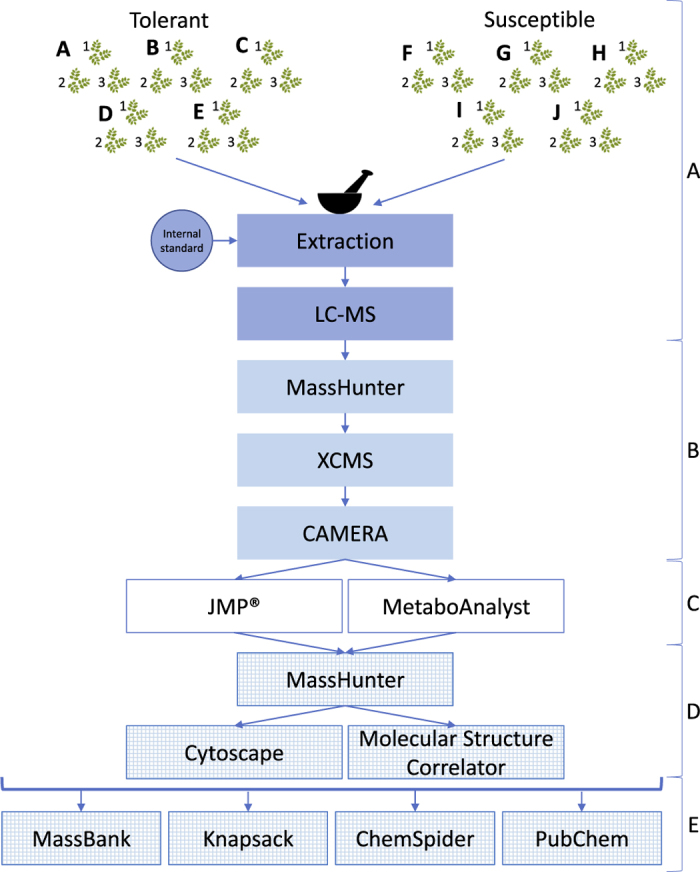
Experimental workflow from sample processing to feature identification. (**a**) Sample processing and mass spectrometry. (**b**) Data extraction (MassHunter), peak identification, alignment (XCMS) and isotope/adduct annotation (CAMERA). (**c**) Statistics (JMP and MetaboAnalyst). (**d**) Fragment ion mass extraction and molecular formula prediction (MassHunter), and feature interpretation(Cytoscape & Molecular Structure Correlator). (**e**) Feature identification (Various databases and literature searches).

These methods are expanded from descriptions previously published in Nature^19^. A schematic overview of the methods are shown in Fig. 1.

### Collection of leaf material

Origin of genotypes used: Leaf material was sampled from trees with very different levels of susceptibility to ADB. Five trees were selected among the individuals exhibiting very low levels of symptoms when subject to natural infection of *H. fraxineus*: R14164C (HGH-A), R14184A (HGH-B), R14193A (HGH-C), R14198B (HGH-D), R14181 (HGH-E) and five trees were selected among trees exhibiting severe symptoms: R14127 (UGH-F), R14120 (UGH-G), R14169 (UGH-H), R14156 (UGH-I), and 25UTaps (UGH-J). All sampled trees were considered unrelated.

The R-trees were selected from a genetic field trial established in 2004 near Randers, Denmark (N56°50, E10°04). The trial comprised 2,448 trees derived from seed collected in 2001 from 101 mature Danish trees. The progenies were monitored annually for symptoms of natural infections from 2008–2011. Material used in this study was derived from selections made in 2012, by which time mortality had increased to 68%, based upon symptom presentation^15^.

The 25UTaps tree was selected among 39 clones based on the average level of crown damage assessed between 2007–2011 across the two Danish test sites at Tuse Næs (N55°45, E11°42) and Tapse (N55°24, E9°27). These represent mature trees selected in Denmark between 1934–1944 (except 25UTap and 27UTaps selected in 1994) and grafted on *F. excelsior* rootstock before establishment in a clonal trial^16^. The level of natural infections in both clonal trials was very high resulting in substantial mortality although all 39 genotypes survive as the clones were originally established with >50 replications (ramets) per clone.

ADB damage of the selected trees was re-assessed in trials in June 2013 and 2014, using the average score to characterise their level of susceptibility (Table 1). Three R-trees sampled as highly susceptible in 2012 were dead by 2013 and by 2016 all susceptible R trees were dead in Randers. 25UTaps is alive and still present in the clonal trials (Tuse Næs and Tapse). All healthy trees retained a 0–10% damage score in 2016, except for R14181 (HGH-E), which had some crown damage (25–50%).

Scions were collected from the trees in either Randers or Tapse during January and February 2013, grafted onto rootstocks of *F. excelsior* seedlings, potted and placed in a greenhouse at University of Copenhagen. Leaves were sampled in September 2014. All plants were symptom free at the time of sampling. Three leaves were sampled from each graft, flash frozen in liquid nitrogen and stored at −80 °C.

### Sample processing

In order to understand whether ash trees with low and high susceptibility to ADB vary in their metabolite profiles as well as their transcriptomes, we undertook untargeted metabolite profiling on a subset of trees of Danish origin from a genetic field trial (R-trees)^15^ and a test panel (25UTaps)^16^. Untargeted metabolomics has not previously been applied to natural populations but has the potential to identify small molecules (or small molecule associations) that directly contribute to tolerance or resistance, particularly given limited evidence for qualitative resistance to ADB and that deciduous trees appear to deploy a combination of constitutive and induced chemical defences. We compared triplicate samples from five low-susceptibility Danish trees (R-14164C, R-14184A, R-14193A, R-14198B and R-14181) and five high-susceptibility trees (R-14169, R-14127, R-14156 R-14120 and 25UTaps). Three leaflets from each triplicate sample were freeze dried and gently crushed to mix tissue types. Approximately 100–150 mg of this material was ground to a fine powder in a 2 ml polypropylene microfuge tube using a TissueLyser (Qiagen; 2×1 min at 25 Hz). 10 mg was extracted in 400 μl 80% MeOH containing d5-IAA internal standard at 2.5 μg/ml ([^2^H5] indole-3-acetic acid; OlChemIm Ltd, Czech Republic), centrifuged (10,000 g, 4 °C, 10 min) and the pellet re-extracted in 80% MeOH. The pooled supernatants were filtered through a 0.2 μm PVDF syringe tip filter (Chromacol, Thermo Scientific, MA, USA).

### Mass spectrometry

These leaf extracts were analysed using a Polaris C18 1.8 μm, 2.1×250 mm reverse phase analytical column (Agilent Technologies, Palo Alto, USA). 5 μl samples were resolved on an Agilent 1200 series Rapid Resolution HPLC system coupled to a quadrupole time-of-flight QToF 6520 mass spectrometer (Agilent Technologies, Palo Alto, USA). Mobile phases were as follows: positive ion mode; mobile phase A (5% acetonitrile, 0.1% formic acid), mobile phase B (95% acetonitrile with 0.1% formic acid). Negative ion mode; mobile phase A (5% acetonitrile with 1 mM ammonium fluoride), mobile phase B (95% acetonitrile). The following gradient was used: 0–10 min-0% B; 10–30 min-0–100% B; 30–40 min-100% B. The flow rate was 0.25 ml min^−1^ and the column temperature was held at 35 °C throughout. The source conditions for electrospray ionisation were as follows: gas temperature was 325 °C with a drying gas flow rate of 9 l min^−1^ and a nebuliser pressure of 35 psig. The capillary voltage was 3.5 kV in both positive and negative ion mode. The fragmentor voltage was 115 V and skimmer 70 V. Scanning was performed using the auto MS/MS function at 5 scans sec^−1^ for precursor ion surveying and 4 scans sec^−1^ for MS/MS with a sloped collision energy of 3.5 V/100 Da with an offset of 5 V. Scan speed varied based on precursor abundance with a maximum of 1.15 s between MS and final (5th) MS/MS (in practice this was never observed to exceed 0.8).

### Data processing

Positive and negative ion data (centroid) were converted into mzData using the export option in Agilent MassHunter. Peak identification and alignment was performed using the Bioconductor R package XCMS^26^ and features were detected using the centWave method^27^ for high resolution LC/MS data in centroid mode at 30 ppm. The samples were grouped into ‘tolerant’ and ‘susceptible’ folders/groups before processing and all samples were aligned and peaks identified in a single batch. Changes to the default parameters were: mzdiff=0.01, peakwidth=10–80, noise=1,000, prefilter=3,500. Peaks were matched across samples using the density method with a bw=5 and mzwid=0.025 and retention time correlated using the obiwarp algorithm^28^ with profStep=0.5. Missing peak data was filled in the peaklists generated from the ADB low susceptibility ash leaf samples compared to the peaklists generated from the ADB susceptible leaves. The resulting peaklists were annotated using the Bioconductor R package, CAMERA^29^. The peaks were grouped using 0.05% of the width of the full width at half maximum (FWHM) and groups correlated using a *P*-value of 0.05 and calculating correlation inside and across samples. Isotopes and adducts were annotated using a 10 ppm error.

### Statistics

Statistical analysis and modelling was performed using MetaboAnalyst v3.028^30,31^, with the following parameters. Missing values were replaced using a (K-nearest neighbour) KNN missing value estimation. Data was filtered (40%) to remove non-informative variables using the interquartile range (IQR). Samples were normalised using the internal standard d5-IAA (POS: M181T1448; NEG: M179T1382). Data was auto-scaled.

**Figure 2:**
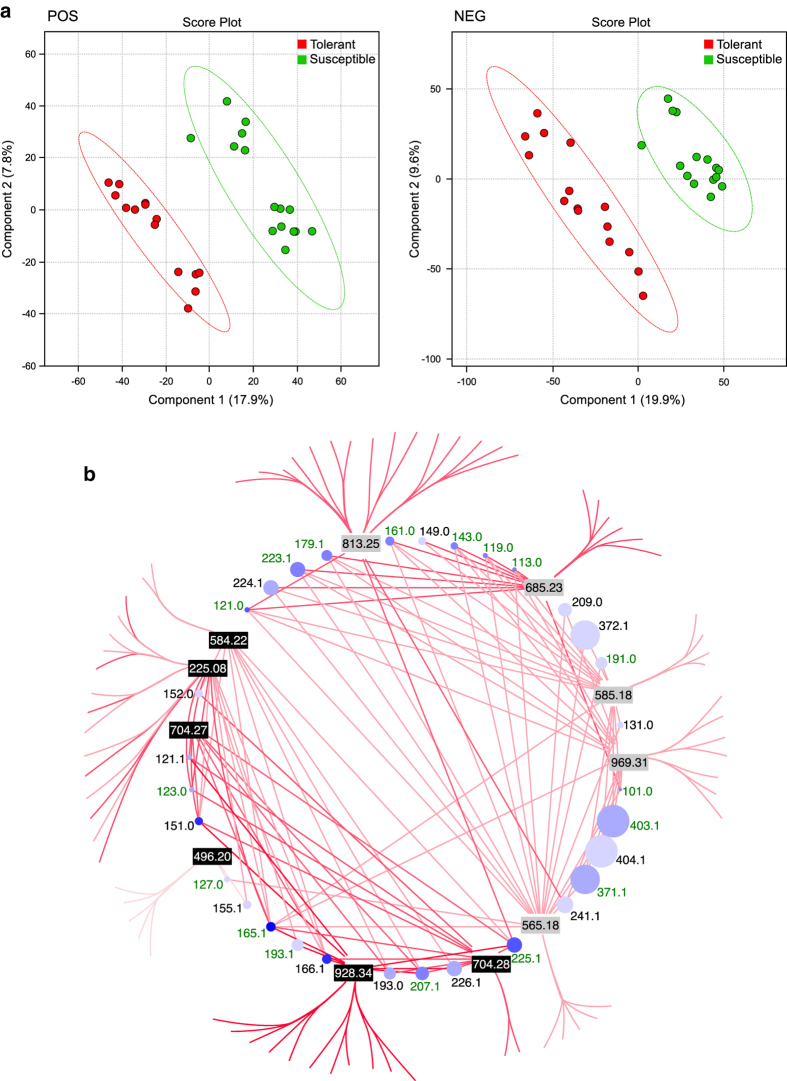
Putative iridoid glycosides as discriminatory features between leaves of *F. excelsior* genotypes with differential susceptibility to ADB. (**a**) Multivariate analysis PLS-DA score plot showing discrimination of five susceptible and five tolerant trees, each replicated three times, based on their leaf metabolite profiles. (**b**) MS/MS fragmentation network of LC-ESI-MS/MS fragmentation data for discriminatory features of high and low susceptibility genotypes. The size of each circle represents the fragment size. The intensity of blue increases as the number of times each fragment is present in all precursor ions increases. The edges are in shades of red based on retention time; the paler the colour the earlier the retention time. The black (POS) and grey (NEG) features are the discriminatory features. The outside ring shows fragments unique to that precursor ion (i.e., not in the other precursor ions). The inside circle is ‘shared’ fragments, present in more than one precursor ion. Those fragment masses shaded in green have been previously reported from fragmentation of iridoid glycosides^41^. Figure adapted from Sollars *et al.*^19^.

Peaks from the three replicates, run in positive and negative mode were aligned with XCMS and features tested for practical significance to determine the differences between the tolerant and susceptible genotypes. In addition, PLS-DA was performed using MetaboAnalyst 3.0 allowing clear discrimination of tolerant and susceptible genotypes based on their metabolic profiles (Fig. 2a).

The individual features (putative metabolites) that contribute to the separation between the different classes were further characterised. We first applied a range of univariate and multivariate statistical tests to determine the importance of these features. This included variable influence on the projection (VIP) values derived from PLS-DA scores, practical significance, *t*-test, *P*-value, Benjamini and Hochberg FDR (False Discovery Rate) adjusted *P*-value, effect size, Random Forest analysis and MS/MS fragmentation network analysis. For example, using Random Forest, significant features were ranked by mean decrease in classification accuracy with 14/15 susceptible samples (OOB error: 0.033; class error 0.07) and 15/15 tolerant samples correctly classified. For all further analyses we chose to use statistical and practical significance (Response screening, JMP version 12) to identify features with a practical significance and validate these using a MS/MS fragmentation network (Fig. 2b). Features that were shown to be of interest using the above statistical tests were individually checked by returning to the raw data and determining if they had been properly identified and aligned by XCMS. We also checked that those features of interest were absent in the blanks and the extraction buffer only samples.

### Putative feature identification

Putative identification of features was performed using several approaches. Each feature of interest was analysed using MassHunter molecular formula estimation. This information, along with accurate mass and MS/MS spectra were used to interrogate existing literature, and databases including, but not limited to, KNApSAcK (http://kanaya.naist.jp/KNApSAcK/), Metlin (https://metlin.scripps.edu), ReSpect (http://spectra.psc.riken.jp/), PubChem (https://pubchem.ncbi.nlm.nih.gov/), ChemSpider (http://www.chemspider.com/), mzCloud (https://www.mzcloud.org/) and Massbank (http://www.massbank.jp/). Additionally, features of interest identified in negative mode were extracted from MassHunter as CEF (compound exchange format) files. These were imported into MSC and searches against ChemSpider and PubChem databases and a custom database using mol files generated from papers. Searches were performed with default parameters apart from the minimum MSC score was set to 50.0. The following parameters were used: MS/MS isolation window was set to first isotope only with default ionization set to protons. Multiple C.E. treatment was set to simple average. Only the 30 most abundant ions were used and formulae contained in the data files was used (i.e., from MassHunter Molecular Formula Prediction). Elements used for MFG composition were H: 2–1,000, C: 1–1,000, N 0–1,000, O:0–1,000 and S: 0–1,000. Height uncertainty was set to 7.5%. Only a representative tautomer was set, the number of total rings and the number of aromatic rings were unconstrained.

### Negative mode feature identification

#### N1

The fragmentation pattern of feature N1 shows a neutral loss of 162 Da (813→651) indicating a possible loss of hexose. Using MassHunter, we predicted the aglycone fragment had a molecular formula estimation of C_30_H_35_O_16_ (diff=C_6_H_11_O_5_) and therefore a potential formula of C_36_H_46_O_21_.

#### N2

N2 also shows a neutral loss of 162 Da consistent with loss of hexose (565→403). The exact mass is close to a secoiridoid glucoside identified in *Ligustrum japonicum* (Oleaceae)^32^ with a molecular formula of C_23_H_34_O_16_. It is also the predicted molecular formula based on isotopic distribution (MassHunter) with a score of 98.44. Although fraxiresinol hexoside has the same mass, the MS/MS fragment sizes are different with abundant peaks of 181 and 373. Another possible candidate is demethylated Excelside A.

#### N3

N3 is predicted to be (7 R/S)-10-Hydroxy-7-methoxyoleuropein (*Ligustrum vulgare* (Oleaceae))^33^, a formic acid [M+FA-H]^−^ adduct of the bitter secoiridoid glucoside Oleuropein/Oleuroside or an acetate [M+CH3COO]^−^ adduct of Demethyloleuropein.

#### N4

Using molecular formula prediction N4 was suggested to be C_31_H_42_O_17_ (score: 99.47). A neutral loss of 685→453 (232) indicates a loss of C_10_H_16_O_6_ followed by a neutral loss of 453→299 (154), which is likely to be C_7_H_6_O_4_. The mass of the molecule is similar to that of Excelside B/Nuezhenide which has previously been identified in *F. excelsior* seeds^34^. Additionally, Nuezhenide has been previously reported to have fragment masses of 453.1389 and 299.1130^35^ which corresponds to m/z peaks of 453.1424 and 299.1108 in the ash leaf spectrum.

#### N5

N5 has a similar fragmentation pattern and retention time to N3 with a mass difference of 384.12 (i.e., C_21_H_20_O_7_). The abundant 403 fragment has the same mass as Oleoside 11-methyl ester/Oleoside 7-methyl ester and shares a fragmentation fragment (223) with these isomers suggests a glycosylated dimer of Oleoside 11-methyl ester or Oleoside 7-methyl ester (((404×2)−1)+162=969)^35^.

### Positive mode feature identification

#### P1

P1a and its M+1 isotope, (predicted by CAMERA to be an ammonium adduct ([M+H+NH_3_]^+^ 686.568) was confirmed by MassHunter as being an ammonium adduct with a formula of C_31_H_42_O_17_ correspond to Excelside B/Nuezhenide. In corroboration, a sodium adduct identified in the practically significant dataset (P1b) and its M+1 isotope, predicted by MassHunter to be C_31_H_42_O_17_ with a sodium adduct. 709→547 represents the loss of hexose. Although the fragmentation patterns of adducts are different it was also detected in negative mode at the same retention time (N4).

#### P2

P2b 589→427, neutral loss of 162 indicates a hexose moiety. CAMERA suggests a sodium adduct, and MassHunter confirms this as a sodium adduct with a predicted formula of C_23_H_34_O_16_ which corresponds to a secoiridoid glucoside from *L. japonicum*^32^, most likely dimethyl-Excelside A, which was also putatively detected in negative mode (N2). There was also an ammonium adduct represented by P2a and its isotope. This is supported by MassHunter as an ammonium adduct with a predicted molecular formula of C_23_H_34_O_16_. As with the Excelside/Nuezhenide feature, adducts fragment differently.

#### P3/4

Both P3 and P4 have a similar mass (m/z: 225.0759, 225.0752 respectively) but elute at different retention times (1,236 and 1,399 s respectively). They have the same predicted formula of 12O_5_ and share some similar fragment ions (95, 151, 165). This could be sinapic acid although based on the spectrum of sinapic acid (http://www.massbank.jp, MCH00015, PR020014, PR101042), the base peak for this compound should be 207. It could be a fragment from an iridoid glucoside, which elutes at the same retention time, some of which have fragment ions with a mass of 225 Da. For example, the ammonium adduct of the Excelside A-like feature P2a which elutes at 1,236 s and the ammonium adduct of the Excelside B-like feature P1a which elutes at 1,398 s all contain fragment ions with a mass of 165 and 151 Da.

#### P5

The isomer of P1, predicted to be Excelside B/Nuezhenide is found as a sodium adduct, P5b, along with its isotope and as an ammonium adduct P5a, although the isomer of the ammonium adduct was not significant.

#### P6

P6b and P6a are sodium and ammonium adducts of the same compound. For the sodium adduct, P6b there is a loss of 162 (501→339) indicating a hexose moiety. The predicted molecular formula of both features is C_20_H_30_O_13_. This is the same molecular formula as the antibacterial phenolic apioglucoside Forsythoside D from *Forsythia suspensa* (Oleaceae)^36^ and kelampayoside A. P6 is possibly an isomer of kelampayoside A as the fragmentation pattern is different to previously reported in *Trachelospermum jasminoides* which had fragment ions of 411 and 369 for the sodium adduct^37^, whereas the *F. excelsior* compound has a base peak of 339.

#### P7

The sodium adduct, P7b and its isotope M929T1536 and the ammonium adduct, P7a and its isotopes M934T1536 ([M+1]) and M935T1536 ([M+2]) suggest a predicted compound formula of C_42_H_54_O_22_. This is the molecular formula of Jaspolyanoside identified in *Jasminum polyanthum* (Oleaceae)^38^ and *Olea europaea* (Oleaceae)^39^. This compound is an aromatic conjugated secoiridoid glucoside and the loss of 162 Da (933→771) is consistent with it being a glucoside.

## Data Records

For this study, we submitted the raw untargeted LC-MS metabolomics data for three replicates of 5 susceptible and 5 tolerant ash dieback genotypes of Danish *F. excelsior* run in both positive and negative mode to MetaboLights^24,25^ (Data Citation 1). Additionally, the XCMS alignment files were deposited (Data Citation 1). The datasets described (Data Citation 1) were previously published in our related work in the journal Nature^19^.

## Technical Validation

### Experimental

Samples were randomised with blanks run after every 5 samples. An aliquot of extraction buffer including the internal standard were run at the beginning (RT=1443.48 s), middle (RT=1442.07 s) and end (RT=1443.78 s) of the run to assess retention time drift. There was a maximum retention time difference of 1.08 s. The maximum retention time difference based on the internal standard in the samples was 3.24 s in positive mode and 7.44 s in negative mode. Retention time values are shown in Table 2. Internal standards had relative standard deviations (RSD) values of 10.6% in negative mode 14.3% in positive mode and ppm differences of −1.3 ppm (negative mode) and −11.4 ppm (positive mode) from theoretical monoisotiopic mass for d5-IAA. Features described in this study had a ppm difference on average of −1.3 ppm difference from the theoretical value with a maximum difference of 5.4 ppm. To assess the experimental error, the mean coefficient of variation (CV) was calculated for each samples in positive and negative mode by using the peak areas of all features reported by XCMS after missing values were imputed by MetaboAnalyst (Table 3).

### Statistics

Several methods were used to validate discriminatory features including equivalence testing, PLS-DA and random forest analysis as described in the methods section.

### Putative feature identification

**Figure 3:**
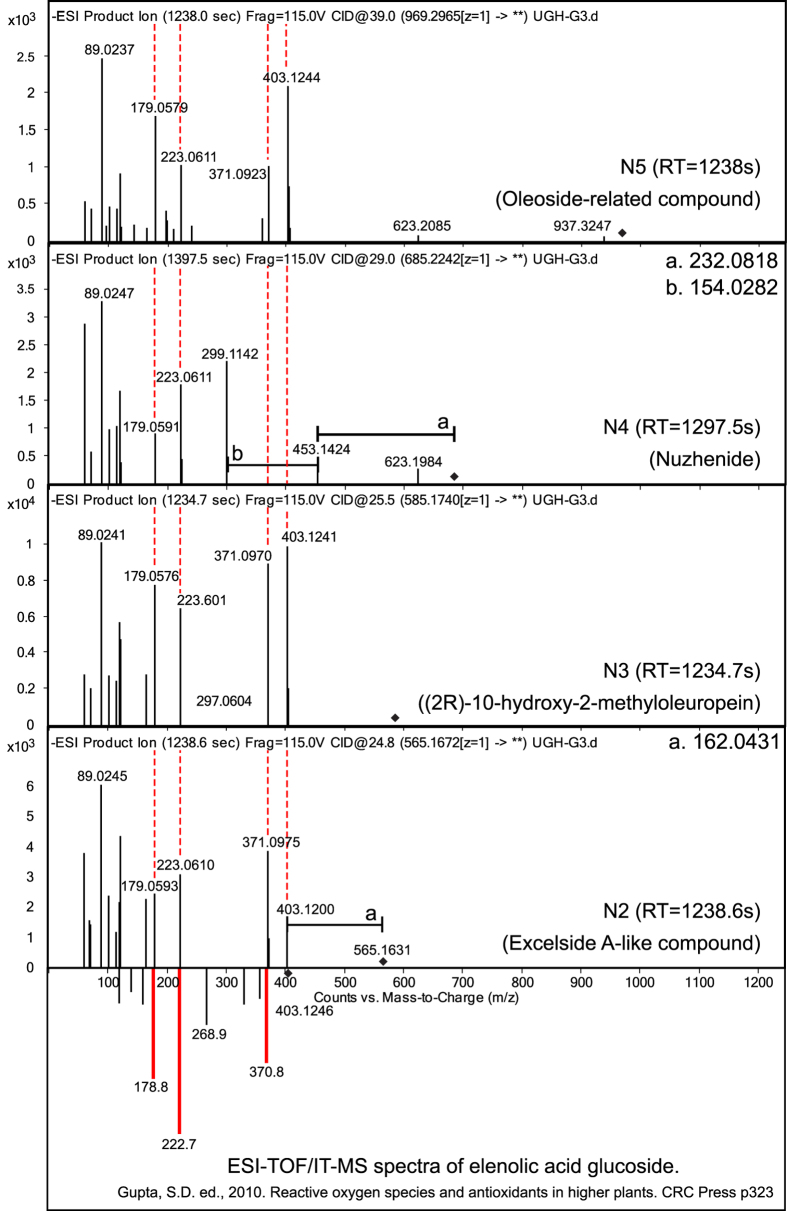
MS-MS Mirror plot of elenolic acid glucoside (ESI-TOF/IT-MS) compared to four negative mode discriminatory features identified in our study (N2, N3, N4 and N5). The spectra share 4 peaks in common, m/z 179, 223, 371 and 403 (elenolic acid glucoside molecular ion). These fragments correspond to a loss of a methyl and hydroxyl group (403–371), loss of hexose (403–223) which is followed by a loss of CO_2_ (223–179). Elenolic acid corresponds to the secoiridoid part of oleuropein-related compounds suggesting that these four features are secoiridoids^42^. Figure from Sollars *et al.*^19^ extended data.

**Figure 4:**
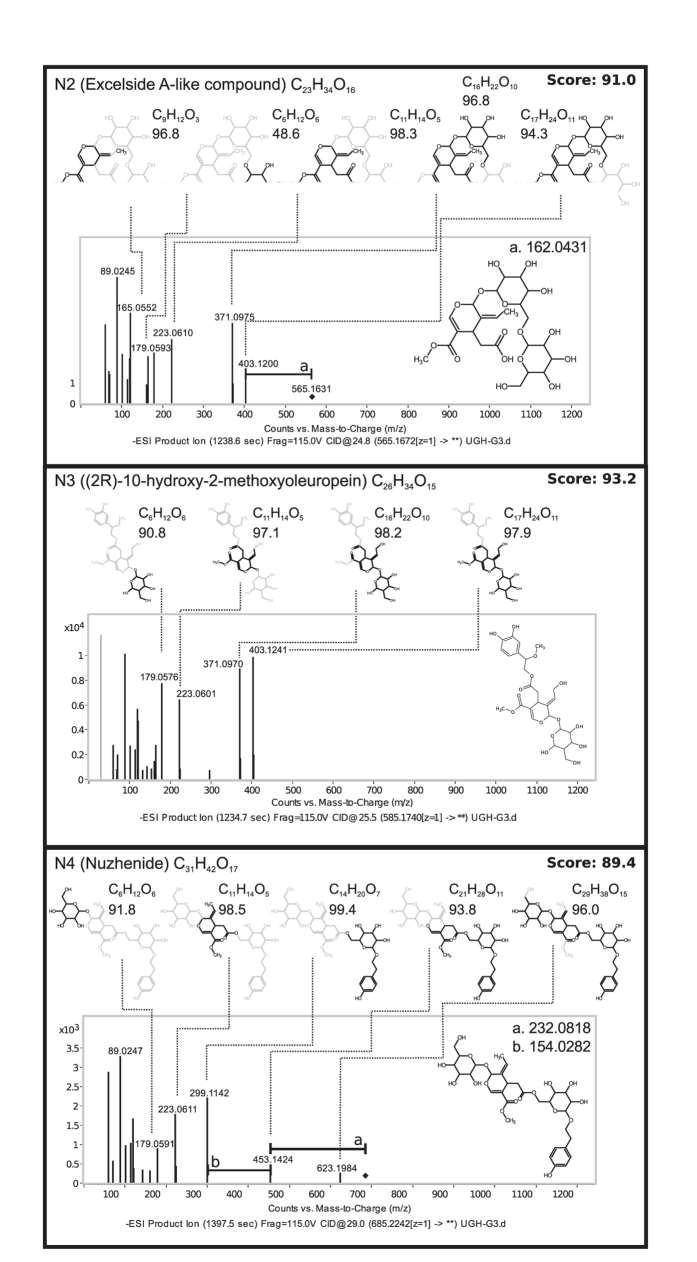
Putative identification of MS-MS fragments for three features observed in negative mode. Predicted structures for three key m/z peaks using Molecular Structure Correlator (Agilent; http://www.agilent.com/cs/library/usermanuals/public/G3335-90176_MSC_QuickStart.pdf) and the structure of putative IDs. Features of interest were compared to the closest known compounds based on molecular formula and accurate mass. The Excelside A-like feature s demethylated Excelside A. The overall score (top right) and individual fragment scores (below molecular formula) obtained from MSC are shown. Bonds and atoms in black are present in that fragment, whereas grey indicates loss. The molecular formula and the MSC score are displayed next to each fragment structure. CID=collision induced dissociation. Figure adapted from Sollars *et al.*^19^ extended data.

Several approaches were taken to validate the putative identification of discriminatory features. An MS/MS fragmentation network was generated after extracting the m/z of the MS/MS product ions (where abundance >5% and m/z >100) from the 13 discriminatory features identified in positive and negative mode using MassHunter Qualitative Analysis Version 4 and visualized using Cytoscape^40^ indicating product ion masses which have been previously reported from fragmentation of iridoid glycosides^41^. Further validation was performed through a mirror plot (Fig. 3) comparing the MS/MS spectra of four features (N2–5) detected in negative mode with an ESI-TOF/IT-MS spectra of elenolic acid glucoside taken from the literature^42^. Finally, the accurate mass of MS/MS product ions from four discriminatory features identified in negative mode (N2-N4) were correlated with the structure of the putatively identified feature using MassHunter Molecular Structure Correlator (Agilent) (Fig. 4).

Identification was not possible for those features with no fragmentation data, or lacking significant supporting adducts. Many additional features of interest were identified but require further validation to allow confident attributions, while some features did not provide fragmentation patterns. We thus restricted further identification and characterisation to discriminatory features from the iridoid glycoside class of compounds. These iridoid gylcosides, which have been previously documented in Oleaceae, are summarised in Supplementary Table S1 and Supplementary Fig. 1. *Fraxinus excelsior* is a member of the Olive family (Oleaceae), therefore data reported for this family were used to aid identification. To further validate our identifications we used an MS/MS fragmentation network approach and graphically illustrate the resulting network in Fig. 2b.

Based on ESI fragmentation patterns, the majority of features identified as having a practical significant difference between tolerant and susceptible genotypes of *F. excelsior* are likely to be from the same class of compounds. Common fragment ions are evident in many of these discriminating features, including m/z 179, 223 and 403, which are indicative of elenolic acid glucoside related molecules. Molecular networking confirms that these features are likely to be from the same family of compounds and we provide a detailed putative identification of each feature that is represented by box plots and spectra (Supplementary Fig. 1).

## Usage Notes

The general approach for sample processing and data analysis is described in the schematic (Fig. 1). Links to the software, resources and repositories used are stated below.

- R: https://www.r-project.org/
- XCMS^26^: https://bioconductor.org/packages/release/bioc/html/xcms.html
- CAMERA^29^: https://bioconductor.org/packages/release/bioc/html/CAMERA.html
- MetaboAnalyst 3.0^30,31^: http://www.metaboanalyst.ca/
- JMP^®^, Version 12: SAS Institute Inc., Cary, NC, 1989–2007: https://www.jmp.com
- CytoScape^40^: http://www.cytoscape.org/
- Molecular Structure Correlator (Agilent):] http://www.agilent.com/cs/library/usermanuals/public/G3335-90176_MSC_QuickStart.pdf
- MetaboLights^24,25^: http://www.ebi.ac.uk/metabolights/
- MassHunter Qualitative Analysis Version 4: http://www.agilent.com/cs/library/usermanuals/Public/G3336-90018_Qual_Familiarization.pdf

## Additional information

**How to cite this article:** Sambles, C. M. *et al.* Ash leaf metabolomes reveal differences between trees tolerant and susceptible to ash dieback disease. *Sci. Data* 4:170190 doi: 10.1038/sdata.2017.190 (2017).

**Publisher’s note:** Springer Nature remains neutral with regard to jurisdictional claims in published maps and institutional affiliations.

## Supplementary Material



Supplementary Figure S1

Supplementary Table S1

## Figures and Tables

**Table 1:** Phenotypes and grafting metadata for samples used in this study.

**Sample ID**	**Tree ID**	**Susceptibility to ash dieback disease (ADB)**	**Year of first grafting**	**Age at time of first grafting (years)**	**Dieback score 2013/14 (% crown damage)**
HGH-A	R14164C	low	2012	9	5
HGH-B	R14184A	low	2012	9	5
HGH-C	R14193A	low	2012	9	5
HGH-D	R14198B	low	2012	9	0
HGH-E	R14181	low	2012	9	5
UGH-F	R14127	high	2012	9	100
UGH-G	R14120	high	2012	9	88
UGH-H	R14169	high	2012	9	100
UGH-I	R14156	high	2012	9	100
UGH-J	25UTaps	high	1997	>50	88

**Table 2:** Chromatographic performance.

**Samples**	**Neg RT**	**Neg RT (s)**	**Neg m/z**	**Neg Order**	**Pos RT**	**Pos RT (s)**	**Pos m/z**	**Pos Order**
Extraction Buffer 1								
Pos—Start					24.058	1443.48	181.1022	
HGH-A1	23.05	1383	179.0866	1	24.06	1443.6	181.0994	1
UGH-F1	23.028	1381.68	179.0867	2	24.072	1444.32	181.1013	2
UGH-G3	23.057	1383.42	179.0868	3	24.034	1442.04	181.1012	3
HGH-E1	23.014	1380.84	179.0869	4	24.043	1442.58	181.1015	4
HGH-C3	23.023	1381.38	179.0871	5	24.086	1445.16	181.1014	5
UGH-G1	23.036	1382.16	179.0862	6	24.064	1443.84	181.1	6
UGH-J2	23.042	1382.52	179.0869	7	24.082	1444.92	181.1012	7
HGH-E3	23.012	1380.72	179.0859	8	24.039	1442.34	181.1007	8
HGH-D3	23.005	1380.3	179.0867	9	24.068	1444.08	181.1	9
HGH-A2	23.007	1380.42	179.0866	10	24.062	1443.72	181.0996	10
UGH-H3	23.014	1380.84	179.0863	11	24.055	1443.3	181.1008	11
HGH-D2	23.004	1380.24	179.0872	12	24.081	1444.86	181.1012	12
UGH-F2	22.979	1378.74	179.0861	13	24.036	1442.16	181.1007	13
HGH-A3	22.995	1379.7	179.0865	14	24.045	1442.7	181.0995	14
UGH-I1	22.981	1378.86	179.0868	15	24.056	1443.36	181.1008	15
Extraction Buffer 2								
Pos—Middle					24.045	1442.7	181.1019	
UGH-J3	23.009	1380.54	179.087	16	24.08	1444.8	181.1009	16
HGH-B1	22.987	1379.22	179.0867	17	24.057	1443.42	181.1008	17
UGH-H1	23.013	1380.78	179.0864	18	24.034	1442.04	181.0999	18
HGH-C2	22.979	1378.74	179.0871	19	24.036	1442.16	181.1011	19
UGH-I3	22.991	1379.46	179.0865	20	24.059	1443.54	181.0994	20
HGH-B3	22.977	1378.62	179.0865	21	24.045	1442.7	181.1002	21
UGH-I2	22.994	1379.64	179.0865	22	24.036	1442.16	181.0999	22
HGH-D1	22.971	1378.26	179.0873	23	24.045	1442.7	181.1	23
UGH-F3	22.951	1377.06	179.0865	24	24.032	1441.92	181.1004	24
UGH-G2	22.951	1377.06	179.0848	25	24.049	1442.94	181.1013	25
HGH-C1	22.933	1375.98	179.087	26	24.049	1442.94	181.1015	26
UGH-H2	22.935	1376.1	179.0868	27	24.038	1442.28	181.1009	27
HGH-E2	22.957	1377.42	179.0869	28	24.068	1444.08	181.1013	28
UGH-J1	22.986	1379.16	179.0871	29	24.075	1444.5	181.1011	29
HGH-B2	22.956	1377.36	179.0861	30	24.044	1442.64	181.1014	30
Extraction Buffer 3								
Pos—End					24.063	1443.78	181.1019	

**Table 3:** Experimental error.

**Tree sample**	**CV % POS**	**CV % NEG**
HGH-A	8.29	6.13
HGH-B	3.83	6.30
HGH-C	4.87	7.51
HGH-D	3.10	9.69
HGH-E	9.54	7.59
UGH-F	12.10	5.71
UGH-G	7.89	2.91
UGH-H	1.51	6.05
UGH-I	6.63	2.36
UGH-J	7.94	1.97
