# Supplementary figures and images for: Ash leaf metabolomes reveal differences between trees tolerant and susceptible to ash dieback disease
Source: Sci Data. 2017 Dec 19;4:170190. doi: 10.1038/sdata.2017.190 (PMC5735976; doi:10.1038/sdata.2017.190)

# POSITIVE

# NEGATIVE

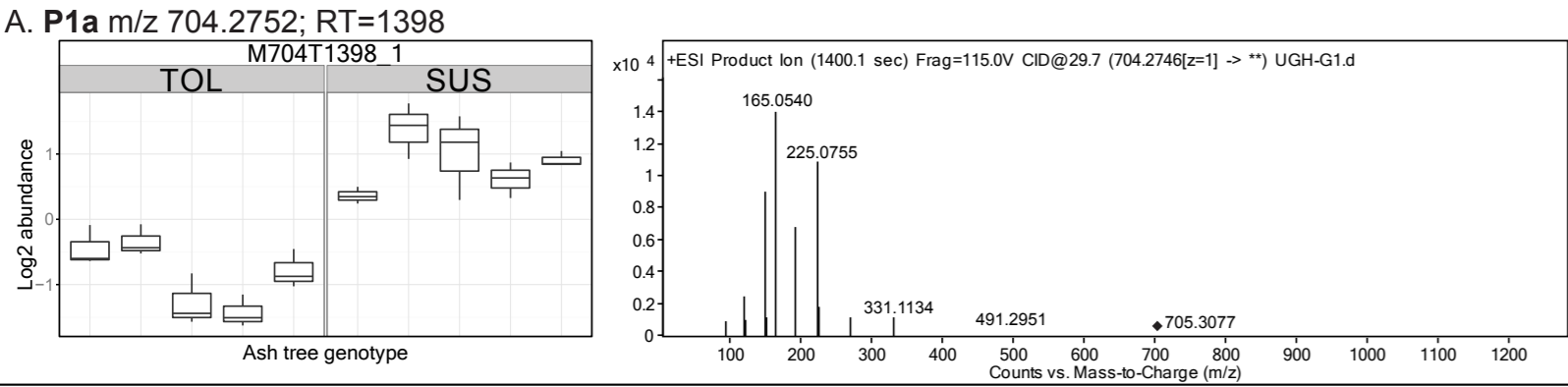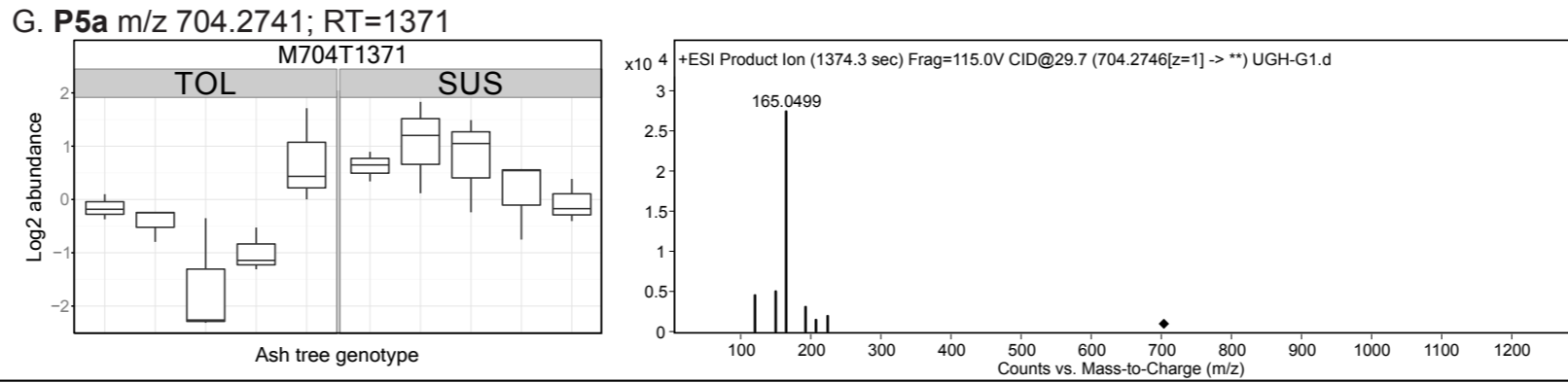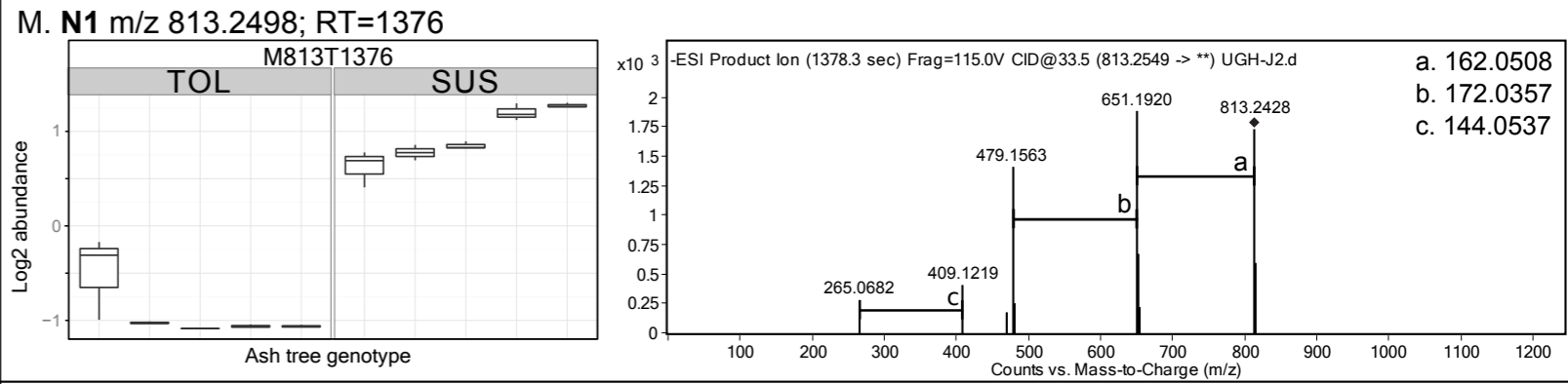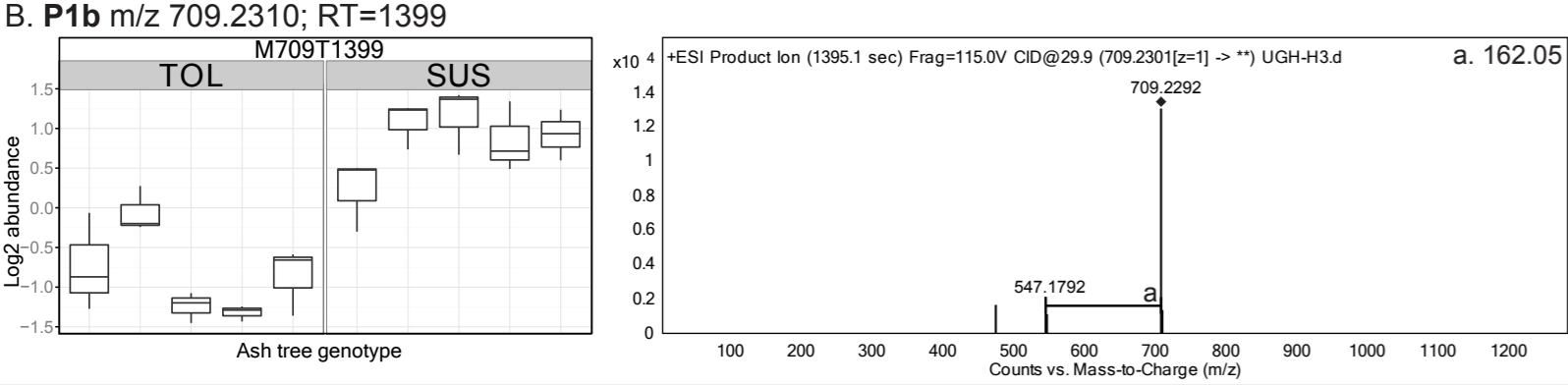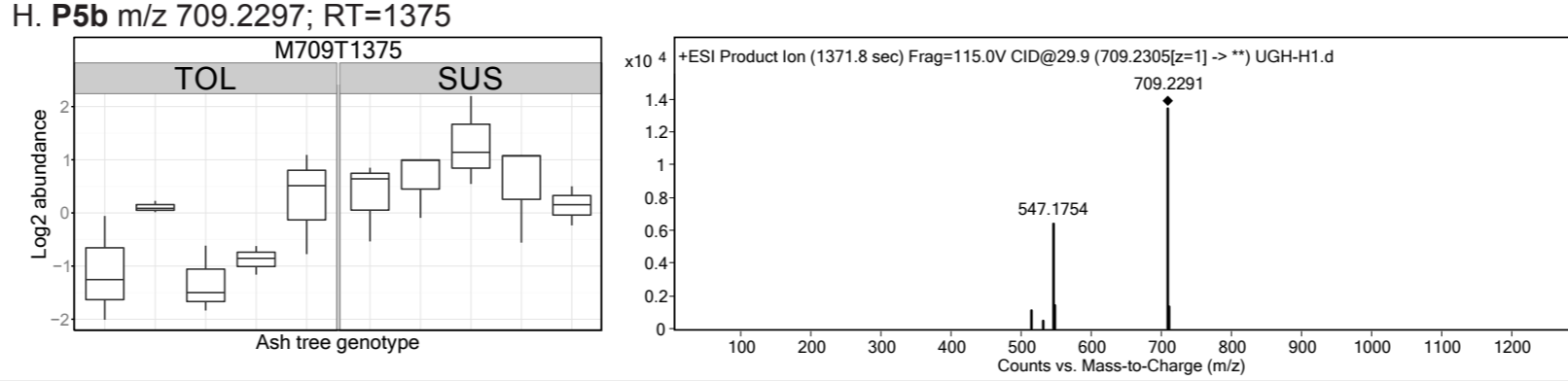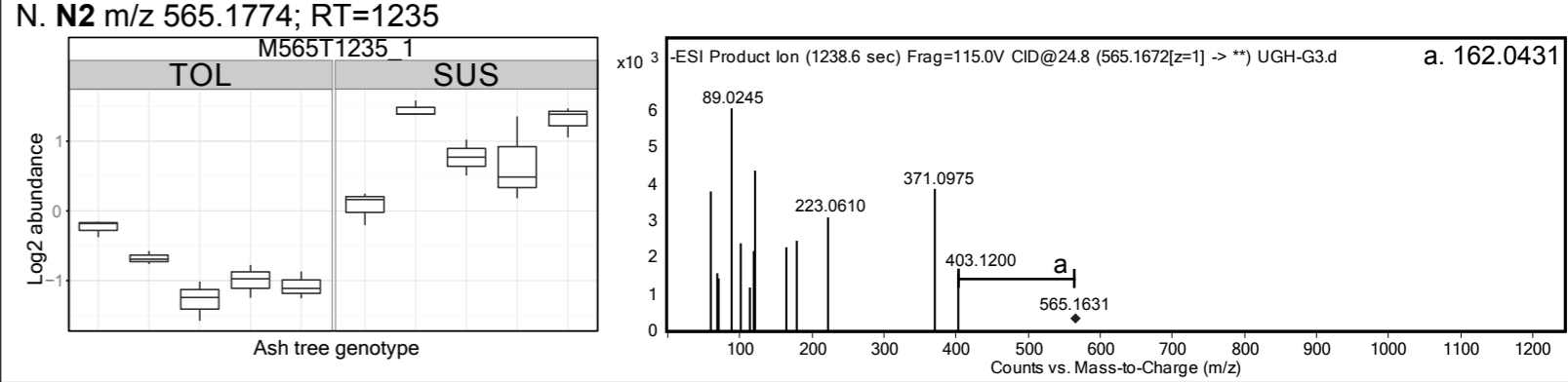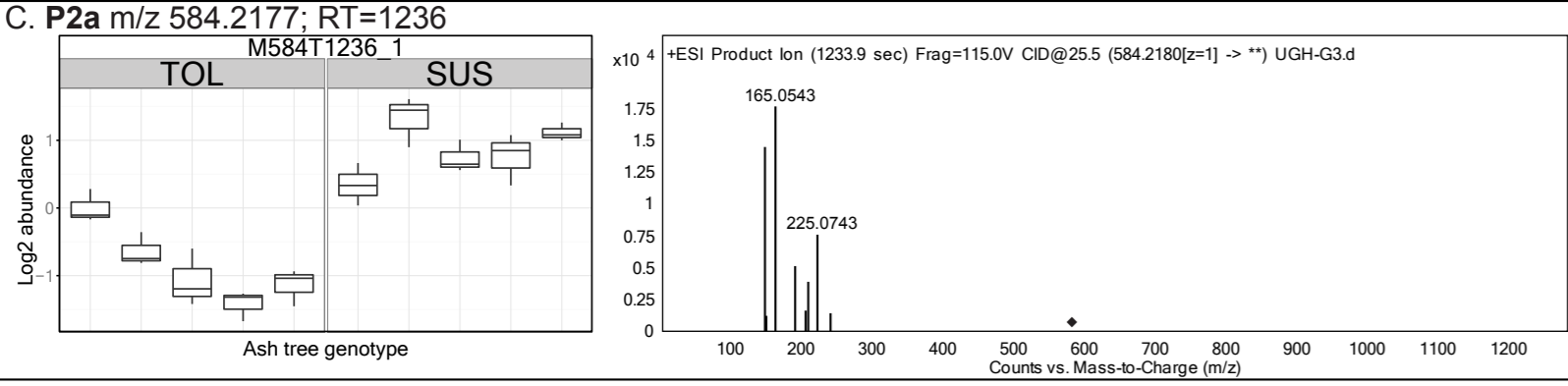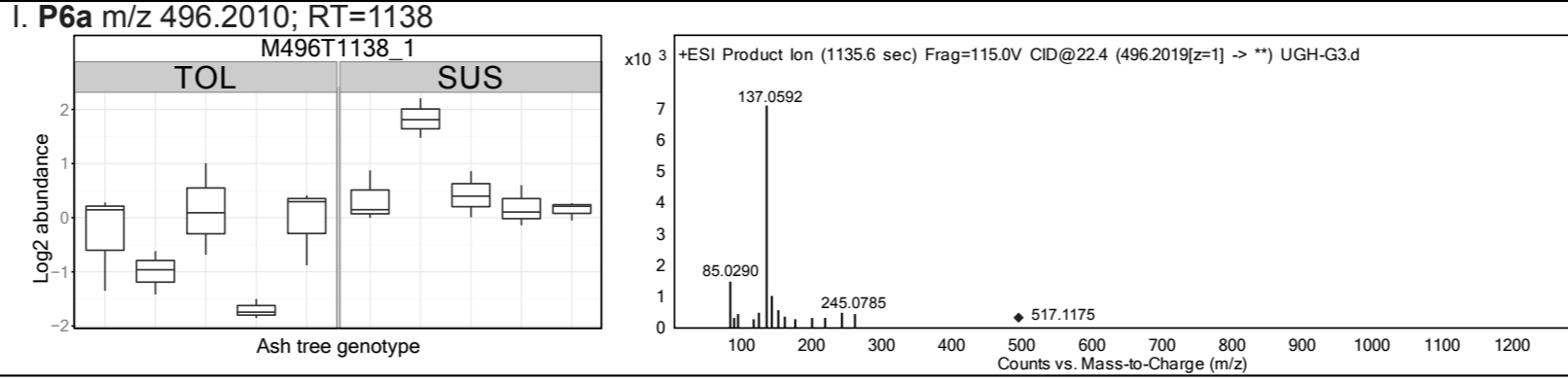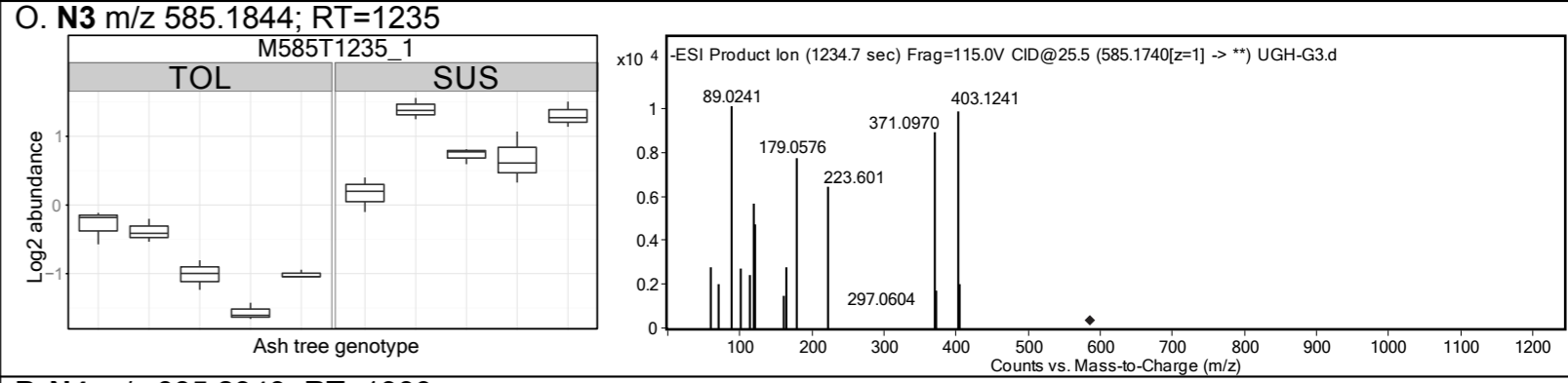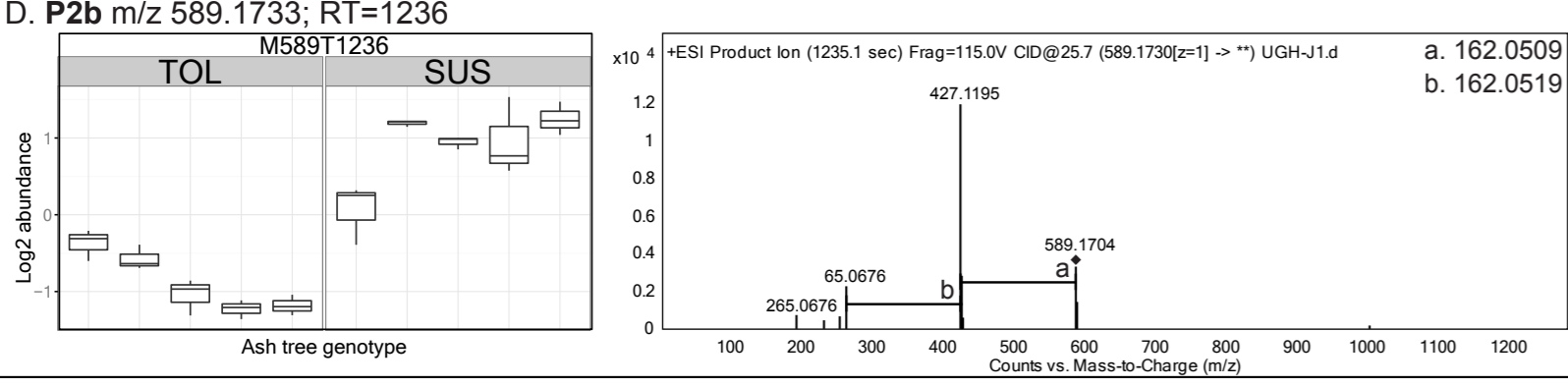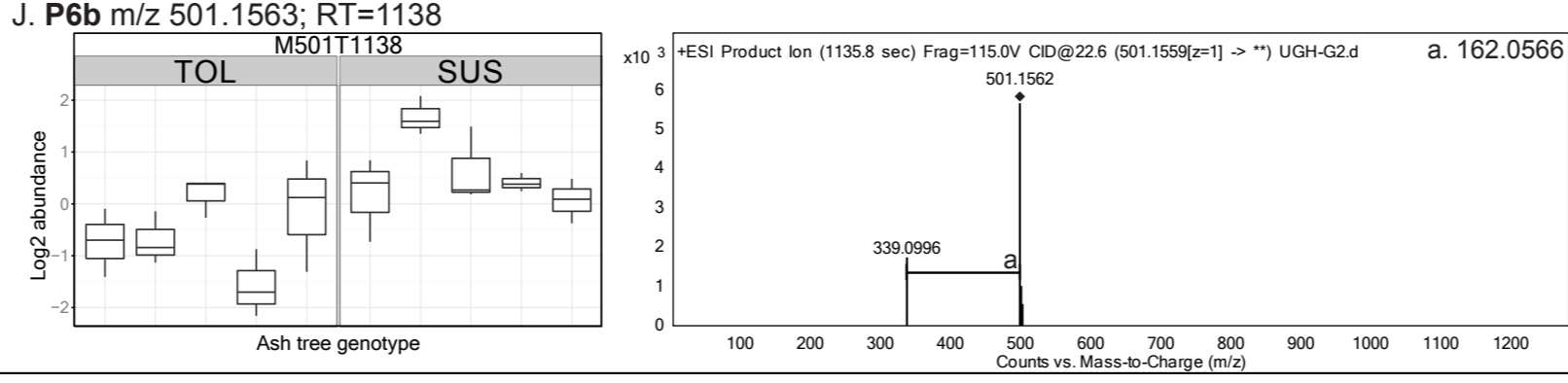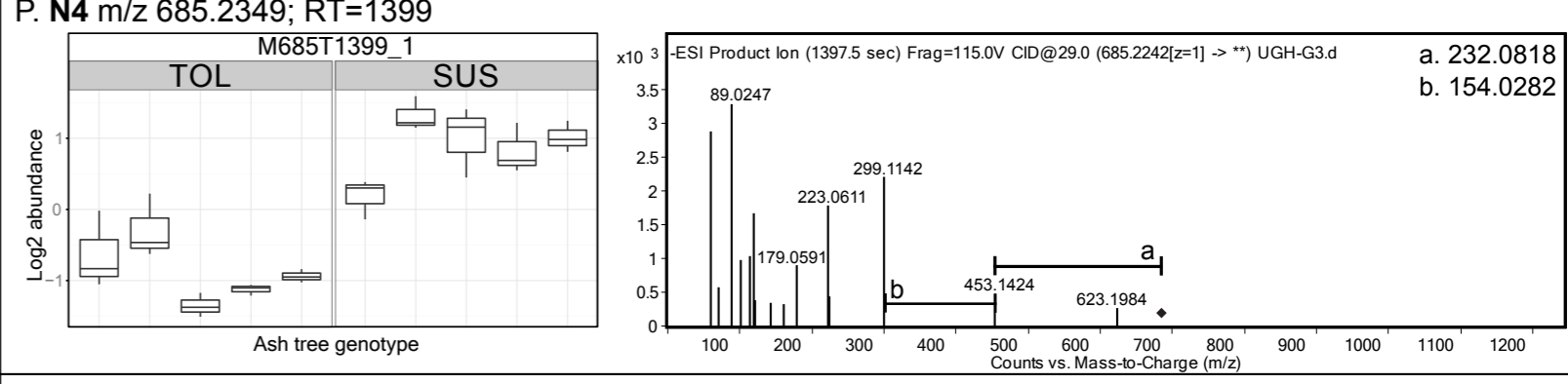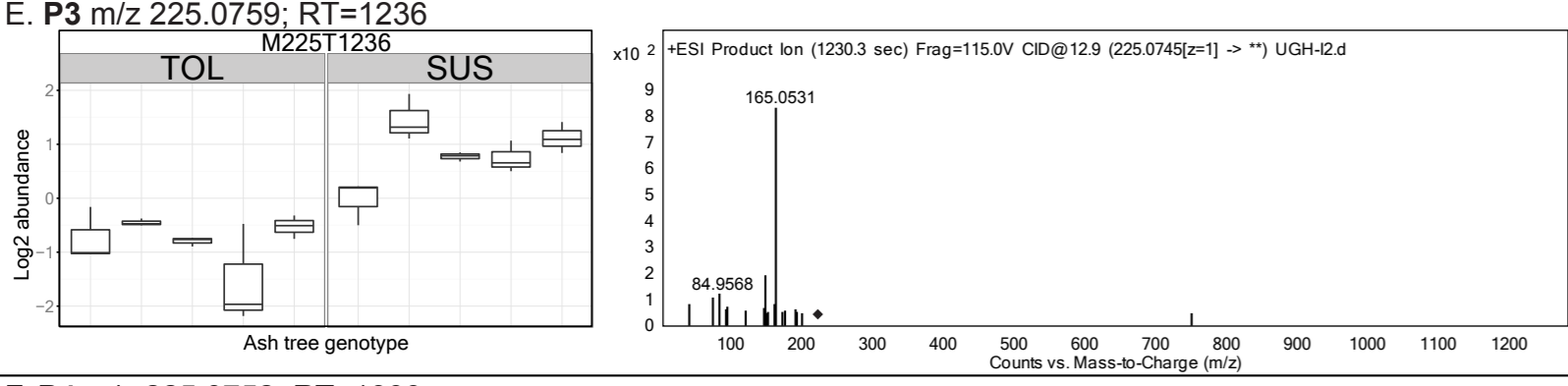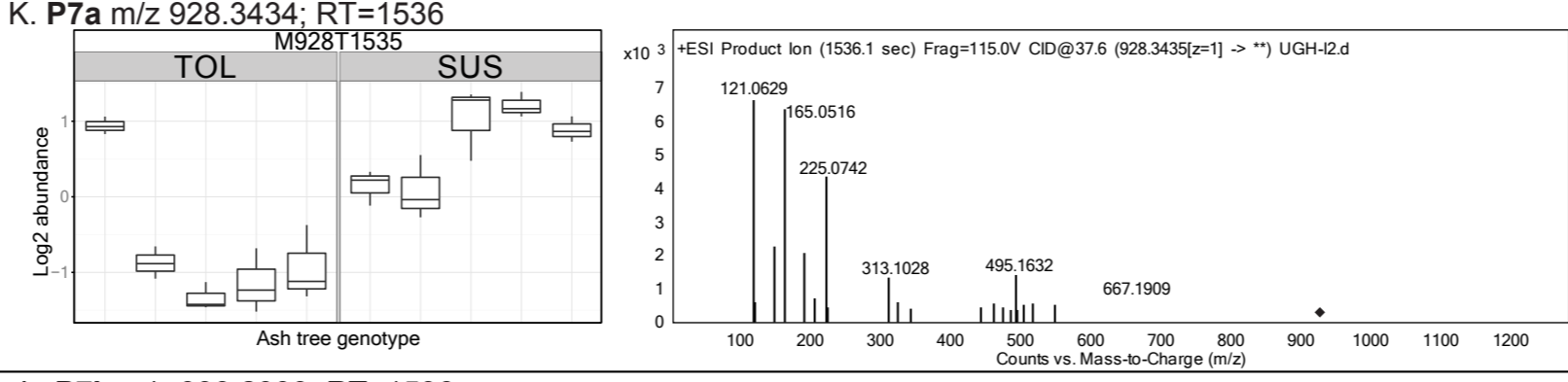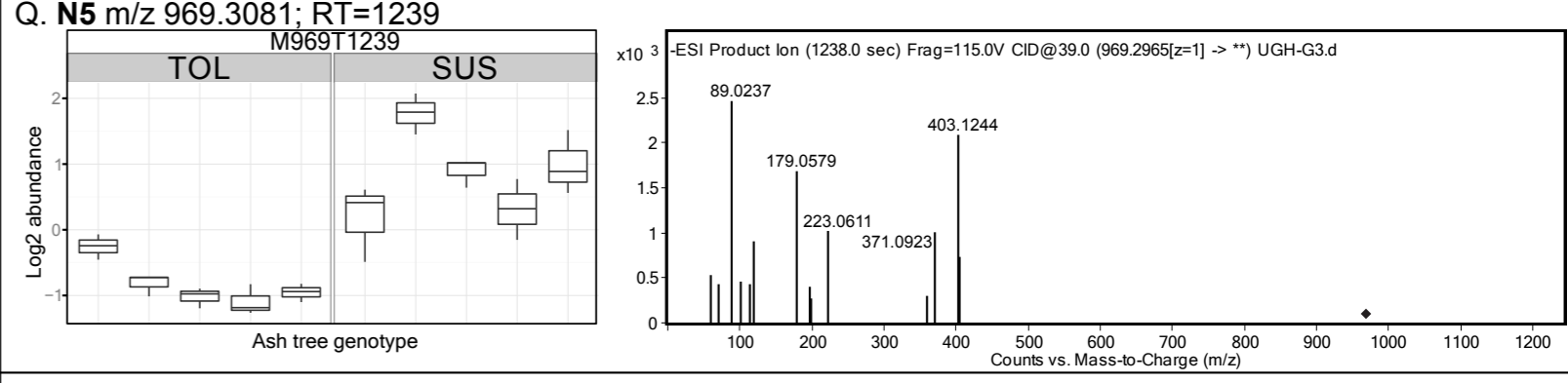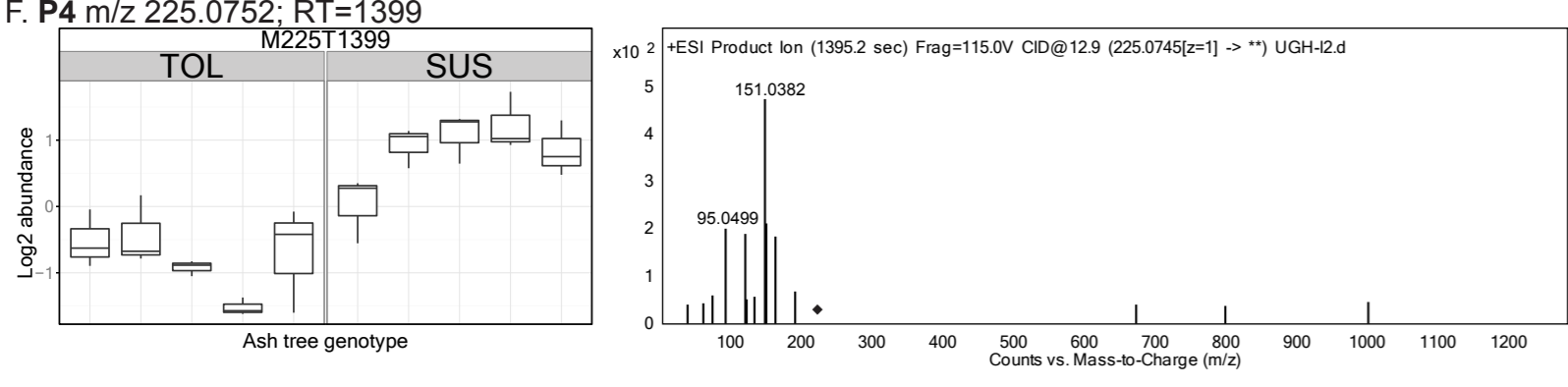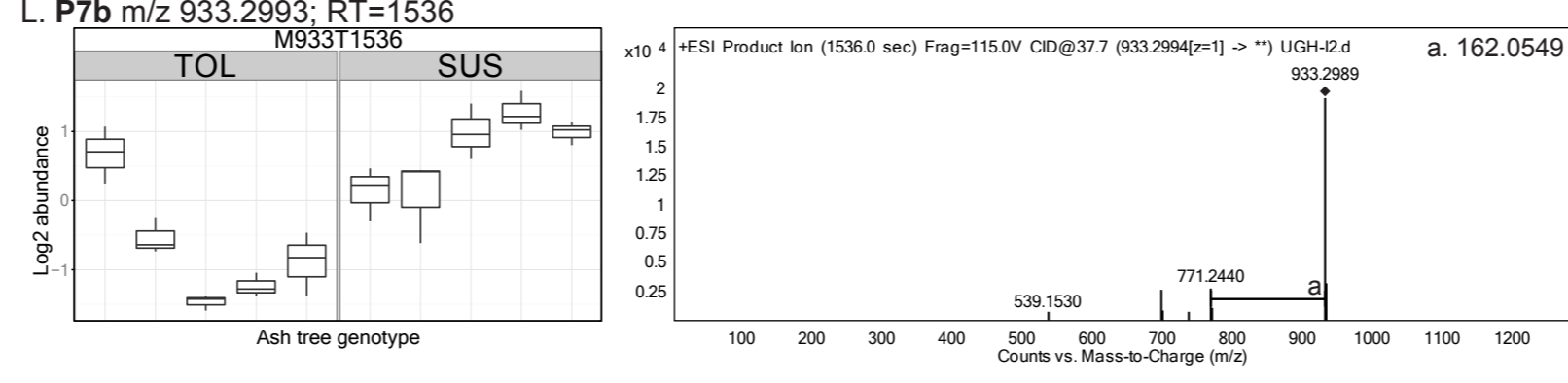

Supplement: Supplementary Figure S1 [file sdata2017190-s2.pdf]
